# Primary care clinicians’ perspectives about quality measurements in safety-net clinics and non-safety-net clinics

**DOI:** 10.1186/s12939-018-0872-3

**Published:** 2018-11-07

**Authors:** Kathleen A. Culhane-Pera, Luis Martin Ortega, Mai See Thao, Shannon L. Pergament, Andrew M. Pattock, Lynne S. Ogawa, Michael Scandrett, David J. Satin

**Affiliations:** 1 West Side Community Health Services, Inc., 895 E 7th St., Saint Paul, MN 55106 USA; 2 Family and Community Medicine, Medical College of Wisconsin, 8701 Watertown Plank Road, PO Box 26509, Milwaukee, WI 53226 USA; 3 Department of Family and Community Medicine, University of Minnesota, 420 Delaware Street SE, Minneapolis, MN 55455 USA; 4 Minnesota Health Care Safety Net Coalition, 1113 East Franklin Ave #202B, Minneapolis, MN 55404 USA

**Keywords:** Primary care quality metrics, Health care inequities, Pay-for-performance, Value-based payments

## Abstract

**Background:**

Quality metrics, pay for performance (P4P), and value-based payments are prominent aspects of the current and future American healthcare system. However, linking clinic payment to clinic quality measures may financially disadvantage safety-net clinics and their patient population because safety-net clinics often have worse quality metric scores than non-safety net clinics. The Minnesota Safety Net Coalition’s Quality Measurement Enhancement Project sought to collect data from primary care providers’ (PCPs) experiences, which could assist Minnesota policymakers and state agencies as they create a new P4P system. Our research study aims are to identify PCPs’ perspectives about 1) quality metrics at safety net clinics and non-safety net clinics, 2) how clinic quality measures affect patients and patient care, and 3) how payment for quality measures may influence healthcare.

**Methods:**

Qualitative interviews with 14 PCPs (4 individual interviews and 3 focus groups) who had worked at both safety net and non-safety net primary care clinics in Minneapolis-St Paul Minnesota USA metropolitan area. Qualitative analyses identified major themes.

**Results:**

Three themes with sub-themes emerged. Theme #1: Minnesota’s current clinic quality scores are influenced more by patients and clinic systems than by clinicians. Theme #2: Collecting data for a set of specific quality measures is not the same as measuring quality healthcare. Subtheme #2.1: Current quality measures are not aligned with how patients and clinicians define quality healthcare. Theme #3: Current quality measures are a product of and embedded in social and structural inequities in the American health care system. Subtheme #3.1: The current inequitable healthcare system should not be reinforced with financial payments. Subtheme #3.2: Health equity requires new metrics and a new healthcare system. Overall, PCPs felt that the current inequitable quality metrics should be replaced by different metrics along with major changes to the healthcare system that could produce greater health equity.

**Conclusion:**

Aligning payment with the current quality metrics could perpetuate and exacerbate social inequities and health disparities. Policymakers should consider PCPs’ perspectives and create a quality-payment framework that does not disadvantage patients who are affected by social and structural inequities as well as the clinics and providers who serve them.

**Electronic supplementary material:**

The online version of this article (10.1186/s12939-018-0872-3) contains supplementary material, which is available to authorized users.

## Background

Quality metrics, pay for performance (P4P), and value-based payments (VBP) are prominent aspects of the current and future American healthcare system, which may impact health disparities [1]. It is recognized that variability in clinics’ quality scores used to compare quality between clinics and between providers can be attributed in part to patient population factors beyond the scope or control of traditional care delivery and patient behaviors, such as poverty, housing, education, and employment [2, 3]. Health care systems and providers serving patients with lower socioeconomic status (SES) or higher burdens of poor structural determinants of health (SDOH) may be disproportionately impacted by P4P, thus widening already evident quality disparities [1, 4].

There are many examples of significant differences in provider quality scores for those serving high and low SES patient populations. In the outpatient setting, primary care providers serving a higher proportion of disadvantaged patients have lower quality scores [5]. Providers in accountable care organizations (ACOs) under Medicare contracts who serve a high proportion of patients with low SES have worse quality scores compared to other ACOs, despite similar practice characteristics and capabilities [6]. Disadvantaged patients [7] and subsequently safety-net hospitals [8] have higher readmission rates. Safety-net hospitals have been disproportionately financially penalized by Medicare’s value-based purchasing and Hospital Readmission Reductions Program [9]. Models have also indicated that Medicare’s Merit Based Incentive Payment System (MIPS) may exacerbate existing disparities due to its focus on specific clinical outcomes with failure to measure other aspects of healthcare quality such as access to care or patient experience [10]. These currently unmeasured aspects of healthcare are often more important to minority and low-income patients [11] as healthcare quality perception differs across race, ethnicity, and language preference [12]. Indeed, Medicare adjustment with a VBP Modifier could lead to exacerbation of racial and ethnic health care disparities due to inequitable payment differences to systems that serve higher-risk and lower- risk patient populations [13].

Current quality metrics do not typically take into account the SDOH factors that can contribute to quality score disparities, and providers caring for disadvantaged populations have greater difficulty meeting quality measures in P4P [14]. Because of this disparity in quality scores, outcomes, and financial penalties, the question of whether or not to risk-adjust quality metrics for socio-economic status (SES) of patients or SDOH risk factors has persisted [15]. P4P appears to have an overall mild positive effect on quality, especially process measures, but the unintended consequences regarding health disparities remain a concern [16]. Although there has been some evidence suggesting P4P may actually narrow disparities for low SES patients [17] and minority patients [18], several studies have indicated that health inequities related to sex, age, ethnicity, and practice type may be exacerbated [16].

While some qualitative studies have explored providers’ viewpoints on quality metrics and P4P [19–26], no studies have included primary care clinicians who have worked in both low-resourced clinics (such as federally-qualified health care systems with generally lower quality scores) and high-resourced clinics (such as private insurance systems of ACOs with generally higher quality scores) in order to understand disparate quality scores in the United States. Our study aims were to identify primary care providers’ (PCPs) perspectives about 1) quality metrics at both safety net clinics (SNCs) and non-safety net clinics (NSNCs), 2) how clinic quality measures affect patients and patient care, and 3) how payment for quality measures may influence health care. These PCPs’ perspectives could be useful to improving quality metric approaches and creating a state level P4P or VBP system.

## Methods

### Setting

Minnesota law requires the Minnesota Department of Health (MDH) to administer a statewide quality reporting and measurement system (SQRMS), and requires Minnesota providers to submit data on these quality measures. Contracting with a private nonprofit organization, Minnesota Community Measurement [27], to gather, report, and publicly publish the quality data, the state uses the data for multiple purposes, including P4P and VBP programs. With the increased use of quality scores for payment and accountability, organizations such as the Minnesota Heath Care Safety Net Coalition became concerned about the substantial influence of non-clinical patient and community factors on provider scores. In 2014, the Safety Net Coalition, the Minnesota Association of Community Health Centers and other organizations formed the Quality Measurement Enhancement Project (QMEP) to conduct research projects in order to account for the influence of SDOH on patients’ health, treatment outcomes, and provider quality scores and in order to influence MDH’s creation of a new P4P system^1^.

This QMEP research project involved obtaining the perspectives of PCPs who had experience working in both SNCs and NSNCs. SNCs were defined as federally-qualified health care centers or Indian Health Services, which serve disadvantaged or uninsured populations and NSNCs were defined as large health care systems or privately-owned clinics which do not routinely offer sliding-fee discount programs for uninsured patients. This QMEP research team included two family physicians (KACP from a FQHC and DJS from University of Minnesota) and three researchers (SLP, LMO and MST) from SoLaHmo Partnership for Health and Wellness, a community-based participatory action research group. The research team created the research design and interview questions, with input from a QMEP Technical Work Group made up of members with expertise in clinical care, quality measurement, research and data. In addition, one of the key informants (LSO a family physician who has worked at both a NSNC and two SNCs) joined the analysis team, in community-based participatory action research fashion [28, 29].

### Design

To identify PCPs perspectives based on their experiences, we chose a qualitative research design with interviews, including in-depth face-to-face key informant (KI) interviews with 4 family physicians to begin our process followed by 3 focus group (FG) discussions with 10 PCPs.

### Recruitment

We recruited all 14 PCP participants by word-of-mouth, emails and snowball sampling; the 4 KIs were identified by QMEP committee members and invited by email; the 10 focus group participants were identified by the KIs, QMEP committee members, SNC medical directors, and invited by email. The two inclusion criteria were (1) primary care clinicians (2) who had worked at both safety net and non-safety net primary care clinics. These were chosen to obtain a diversity of opinions based on PCPs’ experience in two significantly different primary health care settings. Additional 19 PCPs were invited but did not participate (8 were not interested; 8 were interested but could not attend; and 3 did not meet criteria).

### Data collection

Two researcher dyads (KACP and LMO or KACP and MST) interviewed each key informant for 1.5 h, and led three 2-h focus group discussions following the same open-ended question guide supplemented by spontaneous follow-up questions (Additional file 1). In addition, participants completed a demographic questionnaire about their age, gender, profession, race/ethnicity, and work history. We concluded data collection after completing the planned processes that fit our timeline and funds (4 KIs and 3 focus groups), and which coincided with saturation of thematic content and exhaustion of potential participants as identified by our recruitment technique. The University of Minnesota Institutional Review Board determined the study was exempt from IRB overview. Participants were instructed in emails and at interviews that the study was IRB review exempt; were given written information about the study; and were encouraged to keep the information confidential.

### Qualitative and participatory analysis

The audio recorded key informant interviews and focus groups were transcribed verbatim. The 3 interviewers (KACP, LMO, MST) agreed upon a basic organizational coding structure created from the interview questions, forming a framework with which each person then inductively coded each transcript, and then wrote summaries of the main organizational categories. One interviewer (KACP) placed the summaries onto a spreadsheet, following the coding structure. The complete five member research team (KACP, MST, LMO, SLP and LSO) read the transcripts, reviewed the codes and the summaries, discussed codes, reconciled differences, inductively identified the main themes, completed the overall analysis and reached the final interpretation of the data [30, 31]. Three additional QMEP team members joined the writing team (AMP, MS and DJS). The three interviewers (KACP, LSO and MST) selected illustrative quotes to include in the presentation of findings. The participants received copies of the draft report and the article to review; all who responded affirmed the findings and none made suggestions for changes.

## Results

Characteristics of the 14 PCPs are in Table 1. Generally, there are more women than men, mostly older people, mostly family physicians and mostly European-Americans. Results are presented by three themes and three subthemes. Illustrative quotes are in Table 2.

**Table 1:** Characteristics of Primary Care Clinicians

Characteristics	Results
Gender-	
Women: Men	9:5
Age	
Average years (range)	54.85 years, (42–68)
Medical Discipline - N	
Family Medicine	11
Nurse Practitioners	2
Internal Medicine	1
Race/ Ethnicity - N	
European-American	10
African-American	2
Asian-American	1
Latino-American	1
Work patterns in SNCs and NSCNs- N	
Moved from NSNC to 1 or more SNCs	5
Moved from SNC to 1 or more NSNCs	5
Moved from SNC to NSNC to SNC	2
Moved back and forth between SNCs and NSNCs	1

Abbreviations: *SNCs* Safety-net clinics, *NSNCs* Non-safety-net clinics

**Table 2:** Representative Quotes for Themes

**Theme #1: Minnesota’s current quality scores are influenced more by patients and clinic systems than by clinician**	
*Differences in patients at NSNCs and SNCs*	
I think that (NSNCs) have “better patients”...they have middle class people who can do the things that they are asked to do. And they have better health literacy and they have different sets of motivations and priorities. If they are not having to worry about their housing they can probably take care of their diabetes a little better. (KI#2),	
(I)n the populations that we serve (at SNCs)...it’s harder to get patients engaged in their disease processes. And it’s multifactorial...(different) cultural understanding of disease process.... and a (different) culture of patient engagement.... I think it’s a lot harder to engage (our) patients...when you’re trying to survive...all the other things just fall to the side, including management of your chronic disease. (It’s poverty.)…it’s how close is a real grocery store, that’s affordable...how safe is the neighborhood...(KI#1)	
*Differences in clinic structures and processes at NSNCs and SNCs*	
The (NSNC) clinic systems...were quite effective in management of patients with chronic diseases. ..There was an RN…who had the…list of the patients with diabetes but she didn't just tell him to come in--which is what happens now at (SNC) until you end up with like ten patients with diabetes who haven't been seen in over a year—she would go to their medicines and she would. ..adjust medication.... It was very efficient and...she would have them see a PharmD. (FG#1-3)	
A lot of pressure (about QMs in our NSNC). It dominates our meetings always.... You just get all the statistics all the time, and provider spreadsheets of who is at goal, who is not.... There are pool dollars that can be distributed. We are told that how it’s distributed is reflective of your scores...(FG#3-2	
The (SNC) organization doesn’t really push the measures hard. It’s not your (clinician’s) salary depends on that (quality measures). Your pool—the money that NSNCs puts out there (for clinicians)…is not at risk. (KI#4)	
At NSNC system), they encouraged providers to send their difficult patients to that (one) primary care clinic. They encouraged them to do that, so that they can sort of get them all in one setting so that their overall clinic numbers will improve, because if you pull out those outliers... (KI#1)	
*Differences in clinicians at NSNCs and SNCs*	
(T)here’s the whole range of, you know, quality of providers (regardless of SNC or NSNC system they work in).... I don’t (think SNCs clinicians are reason for lower quality scores). I think, if anything, our providers are more activated to try to comply with the measures.... Generally people are very engaged, and they want to do better and have the patients do better as well. (KI#2)	
**Theme #2: Collecting data for a set of specific quality measures is not the same as measuring quality healthcare**	
*Perspectives about measurement*	
You know that famous statement by Einstein: "Everything important can't be measured."... So when I thought about this, I thought about trust and about how do you get patients from a different culture to trust you?...but it's a two-way street so the provider also needs to trust them.... I thought: but it starts with the patient. They'll think it's quality if you...really care about them...and then you really have to just accept them for whoever and where they're at. And just be in that place with them and go along together them and then they'll think this is quality because this is someone who (cares)…. And I think it's the relationship that matters to them. And then, as that is established, then you have more influence... and then you can try and get them do things that they might not want to do....and that it is truly patient-centric and not doctor-schedule centric. (FG#2-3)	
*QMs are valuable*	
(S)o I think they (quality measures) are important because they do help improve (care), at least we think they help improve, health ... (KI#3)	
(T)here should be systems in place that make sure that people aren’t truly falling through the cracks…(KI#1)	
*QMs are not valuable*	
P1: (At NSNC), that's where that big push in your 160 person lists is...a medical assistant checks these things when you come in (weight counseling)...and you check the box you had counseled them (unhealthy living).... You check the box or hand them a piece of paper (tobacco counseling). Um, I thought some of those things well were not very meaningful. P2: It was meaningful to the business people running the show. P1: The boxes were checked. P2: That's what mattered. [group agreeing] P3: And again, it gets at the measures themselves, but what are really the expectations of the measures? (FG#1-1, #1-2, #1-3)	
*Responses to competition about QM scores*	
To be honest, I was very competitive (at NSNC).... I was personally kind of motivated to say we need to beat them (other clinics in same system).... I was also kind of the cheerleader that the other physicians could get behind and I kind of drew them into some of the competition...(KI#3)	
At [NSNC] a larger and larger proportion of compensation is aiming to be based on quality numbers. And there was…a difference of...take home (pay) at the end of the year which made people really mad.... (Some) were much more driven by money and productivity.... (Others) would look at the sheet and toss it aside and take care of their patients however they wanted to. But when it comes to, you know, $30,000 at the end of a year, you get kind of crabby. (FG#1-1)	
At (NSNC), they broke it down by provider and they kept saying, we’re breaking it down by provider because it shouldn’t matter, the population that you’re taking care of. So I had trouble to begin with because I think it does matter who you’re taking care of. And when they would do that then, then it would be very punitive. And that’s where the problem, I think that’s not a good thing. Because it shouldn’t be punitive. It shouldn’t be punitive for the provider and it shouldn’t be punitive for the patient either. And it’s both. (KI#1)	
I found that the people (clinicians at NSNCs) who were high-performing were long-term part-time and they had a small population, so their percentages were very high. They didn't do a damn thing. They didn't have hard patients. They didn't have long days. They didn't take new patients. They were closed practice--that's another issue. If you had a closed practice, you stabilized that group. You know, if you were fortunate, a lot of these guys they were smart enough that they chose their population. They knew something was coming. They dumped their bad patients and they kept the ones that were good. I mean duh we weren't that stupid that we couldn't see that. (FG#1-2)	
*Financial costs of QMs*	
(Percent administrative cost is) way too high....(A NSNC system) is investing way too much in getting these numbers better without really improving healthcare...without adequately improving quality, I would argue that. And not just that…those funds are being shifted…so that money is being shifted (away from other aspects of care.) (KI#1)	
Why do we have this kind of competitive sense of things? Are we really making that much more money if we have better scores?... Are we elevating the quality of our communities? That’s what I want (to know). (FG#2-1)	
**Subtheme #2.1: Current quality measures are not aligned with how patients and clinicians define quality healthcare**	
*Patients do not know about QM scores*	
I’ve never heard, even in (Suburb at NSNC), I’ve never heard one person say ‘I chose your clinic because I read Minnesota Community Measures’ (scores).... (P)eople choose clinics and physicians ... for many reasons but I don’t think online reviews or quality ratings are one of them. (KI#3)	
*Patients define quality of care differently than QMs*	
P2: And there's a lot of mistrust. There's a lot of: “you talking over my head so you don't care about me”.... I hear that all the time..."Do you care about me?"..."Really, do you care?".... But it takes a long time. It takes a long time to develop those relationships. P1: ...I think to them it's (quality) just completely different (from quality scores), like "Do you know my history when I walk in the room? "Do you know what's going on with me and are we picking right up where we left off the last time?.... It’s...“if I feel I've been heard, you care about me”, that's quality to my patients. P2: (T)hat's what I'm saying. It's that sense of: “you know me, I trust you”...and I really take that seriously. (FG#2/1, #2-2)	
*Clinicians define quality of care differently than QMs*	
Quality (is) so much more complex than this (quality score) could ever get at, for me. Because quality is, for me as a family doc, quality is continuity in care. It’s that I know my patient. That they know me. That they trust me. That I trust them. That we have a combined working relationship. That I give them what I can offer, but that they take responsibility for what they can offer. (KI#1)	
I feel that quality is just moving someone into the right direction, whether if it’s the endpoint or not, just getting them to head in the right direction is already quality for them and for me. It’s good to know that you’re doing something without having those objective numbers at the end. Relative improvement towards various health goals for them to feel good about themselves and their health and the decisions that they’re making (is what quality healthcare is to me). (FG#3-3)	
**Theme #3: Current quality measures are a product of and embedded in the social and structural inequities of the American health care system**	
*QMs are based in social inequities*	
All of this (quality measurement) has some political aspects…at various levels…and certainly the pharmaceutical industry has run research and guidelines in the US for generations. (KI#2)	
(P)eople that are in Minnesota, it’s still pretty white folk land and the decision makers are still coming from that heritage. And they don’t have...an understanding that other people live differently than they do. (KI#2)	
*QMs measure unequal processes*	
I think one of the troubles we have is (that) many of the measures and the programs and much of what comes from the state is coming from an upper-or middle-class perspective. People who have resources, who have insurance, who have the means, the wherewithal, the transportation to do the kinds of things they need to do, to take care of diabetes, for instance, better. When people don’t have those things then they experience barriers to that care.... And what doesn’t seem to get lots of lip services at the DHS (Department of Health Services) about...is social determinants. (They say;) Oh yeah we get it. (But I say:) Oh no you don’t! ‘cause you have not changed how you’re approaching this whole process (of measuring quality care). (KI#2)	
**Sub-theme #3.1: The current inequitable healthcare system should not be reinforced with financial payments**	
*Finances tied to QMs*	
I want to make sure that...at least we (NSNCs and SNCs) are all on the level playing field, and we've never been that way, and that's just the nature of our practice so…we (SNCs) shouldn't be penalized, because we have a diverse population that no one else wants to care for.... (FG#2-2)	
It’s not fair (P4P based on quality metrics). You’re penalizing the clinics that are trying to work with people and do the best they can, from where they (patients) are coming from, for the insufficiencies of people’s real lives in the real world that don’t conform to what somebody has decided is what they should do and then we (the clinics/ physicians) are being penalized for that? It’s not fair...because you know: no money no mission. (KI#2)	
**Sub-theme #3.2: Health equity requires new metrics and a new healthcare system**	
*New metrics*	
I think anyone in poverty already has a level of complexity that they start with....We need some way of recognizing that (when choosing measurements). I think part of it is figuring out the (quality) services and not docking the organizations that are trying to help these people.... You want to reward the good, but you don’t want to dock people who aren’t meeting these. (FG#3-3)	
I feel that quality is just moving someone into the right direction, whether if it’s the endpoint or not.. (We could measure) relative improvement towards various health goals for them to feel good about themselves and their health and the decisions that they’re making. (FG#3-2)	
*New healthcare system*	
That’s foundational to quality: give everybody health insurance and cover their medications, all their chronic medications should be covered. And then there’s all these other stuff like outcomes (that we have to deal with), but at least get that off the table. (FG#3-1)	

*Abbreviations: KI* 4 Key Informant PCPs: #1, #2, #3, #4, *FG* 10 Focus Group PCPs: #1-1, #1-2, #1-3, #2-1, #2-2, #2-3, #3-1, #3-2, #3-3, #3-4

### Theme #1: Minnesota’s current quality scores are influenced more by patients and clinic systems than by clinicians

Participants view disparate scores at NSNCs and SNCs as being due to differences in the patient populations who attend these clinics and due to variations in the clinic systems that support clinicians. Differences in patient populations lead to disparate quality metrics between NSNCs and SNCs. Minnesotans who attend NSNCs are seen as being more able to act in concert with the quality measures because they generally have low burden of SDOH, have health insurance, and have literacy levels, education, and cultural backgrounds that are generally congruent with mainstream medical culture. Thus, they are more capable of engaging with the clinic-based efforts to respond to quality metrics, especially the bio-medically defined self-management processes that are necessary to improve quality scores of chronic diseases.

Generally, Minnesotans who attend SNCs have higher burdens of SDOH, which pulls their energies and resources away from health and health care. Many are uninsured or under-insured, have low English proficiency, have low medical literacy, are immigrants or refugees, and come from diverse cultural backgrounds that are less in concert with, or even in conflict with, mainstream healthcare culture. Cultural issues due to differences in language, expectations of the role of healthcare systems, health care’s focus on individuals rather than on families, and cultural concepts of health, healing, and decision-making, as well as historical distrust and discrimination, influence the incongruence.

Differences in clinic systems lead to disparate quality metrics between NSNCs and SNCs. NSNC health care systems use their higher financial resources to create clinic teams, clinic workflows, electronic medical record (EMR) processes, and adjunct patient education approaches that specifically address the metrics, as administrators view the metrics as promoting cost effective care.

In addition, NSNCs have financial and social incentive programs to influence clinicians to act on the metrics in order to increase their quality scores, so that clinicians will address the quality metrics in their interactions with patients and the EMR. In contrast, SNCs do not have the financial resources to develop teams, workflow processes, and systems specifically aimed at metrics. Most have not created the quality report cards or implemented financial incentives aimed at improving clinicians’ quality scores. Also, clinician and staff energies are diverted from quality metrics to deal with other aspects of patient care (language, health literacy, medical-legal-social issues, etc.).

There are specific clinics that serve low-income or immigrant populations within NSNC systems. Generally, these clinics have lower scores than other clinics in their systems because the clinics serve patients with high SDOH, and have higher scores than SNCs because they have more system resources. It is in these clinics that individual providers feel the punitive nature of linking quality scores with job performance and financial remuneration, since their colleagues in other clinics with patients with low SDOH burdens have better scores.

Overall, these PCPs do not see the differences in quality scores between NSNC and SNC as being due to clinicians’ having variable knowledge, skills, and abilities.

### Theme #2: Collecting data for a set of specific quality measures is not the same as measuring quality healthcare

These PCPs see value in quality measures, but assert that healthcare quality measures should not be conflated with measuring quality healthcare. Quality measures are valuable when they are consistent with professionals’ mission to improve people’s health, when they provide clinicians with population-based perspective, when they are based on evidence-based medicine, and when staff and systems assist the PCP in providing care, which can prevent patients from “falling through the cracks”.

However, quality measures are not valuable when they result in clinicians’ taking empty actions that are “just clicking boxes”, when measures are impossible for their patients to meet, when they take clinic visit time away from connecting with patients’ focus on health problems, and when their actions improve scores but do not improve patients’ health.

In addition, quality measures can harm care, as clinicians focus their attention on things that are measured rather than things that are not measured, as they “cut corners” in order to avoid being overworked, work more hours to “click more boxes” (which has contributed to professional dissatisfaction and burnout), shuttle low-scoring (or “non-compliant”) patients to other providers or other clinics, take financial hits to their base salaries, or reduce their clinic hours (thus decreasing access), or adjust their practices in order to keep “high-performing patients” so their scores are good.

Specific clinic processes that focus on increasing quality scores include public displays of clinician specific data within clinics and clinic specific data within large healthcare systems. A few participants feel the positive nature of data displays and competition between individual providers, teams, and clinics. Most participants express discontent with the negative consequences of publicly displayed data and tying compensation, performance review, and even termination to quality scores, calling these tactics “shaming”, “unfair”, and “punitive”. These processes feel unfair because of the inequalities of providers’ patient populations, which lead to inequities of provider workloads and ability to meet measures. Finally, some participants argue that the process to improve quality measures and the money used to build the processes are not being used wisely to improve health.

### Subtheme #2.1: Current quality measures are not aligned with how patients and clinicians define quality healthcare

Participants sense that most patients are unaware of current quality measures, their scores, their doctors’ scores, or their clinics’ scores. They speculate that some patients from high socio-economic class backgrounds who attend NSNCs may be aware, but not those who attend SNCs. They sense that for most patients, the current metrics do not measure the important aspects of quality healthcare, as patients would not define quality healthcare by numbers, cut-off values, or percentages of numeric goals; for most patients, these are too remote from their lived experiences. Rather, patients would define quality healthcare by their subjective sense of well-being, feeling respected by their doctors, nurses, and clinics, and being in trusting relationships with clinic staff and PCPs.

Clinicians define quality health care in broader ways than in the narrow, specific and well-defined quality measures. Their definitions include philosophical aspects of relationship-based care, from listening to and caring about patients, educating and empowering patients, having a therapeutic relationship with people, and developing a patient-provider partnership that helps people to attain their personal health goals. In addition, they stressed that patients should define their own health goals in whatever way is important to them, such as quality of life and satisfaction with their lives.

### Theme #3: Current quality measures are a product of and embedded in the social and structural inequities of the American health care system

Participants express the view that the current quality metrics are based on social inequities. Many of the measures are grounded in inequitable research that gave rise to evidence-based medicine, whose data was generated from population studies done on majority white Americans. As such, they are not based on data collected from other specific patient populations.

Some people further identify the quality measures as unequitable tools that historically were selected by inequitable social processes*.* Initially, metrics were chosen by corporate executive officers who were purchasing health insurance as a technique to evaluate the quality of the product in balance with the cost in order to make wise financial decisions. Then, healthcare organizations and insurance plans adopted them to improve quality health care while curtailing costs, and government officials chose which measures to use to compare clinic and clinician performance in order to reduce costs. Current traditional quality measures have not been selected with input from patients or from communities living with the highest levels of disparities in health.

The quality metrics measure unequal processes. The disparate results between SNCs and NSNCs reflect the privilege of the insured, educated, middle and high social-economic class white Minnesotans whose lower SDOH burden and congruence with biomedical systems contribute to their higher quality scores. The current quality measurement system quantifies the biomedical view and the hierarchical American society into a “quality score” that shows lower class people at the bottom and higher-class people at the top, in congruence with social inequities.

### Sub-theme #3.1: The current inequitable healthcare system should not be reinforced with financial payments

While acknowledging that the healthcare system is changing to P4P and VBP processes and concrete metrics are a necessary component to that process, participants express concern about the inequity of a clinic-based financial payment system that is tied to quality scores. Inequality in healthcare and healthcare quality measures mean that the neediest clinics serving the neediest patients will receive the least amount of money, when, in reality, they need more money to respond to patient population health needs.

### Sub-theme #3.2: Health equity requires new metrics and a new healthcare system

These PCPs emphasize the need to: 1) operationalize patient-centric definitions of quality, beyond patient satisfaction, which are based on patients’ health goals, patients’ healthcare experiences, and patients’ assessments of the quality of their relationships with clinicians; 2) implement clinic processes to expand the team, hire staff from diverse communities, and have adequate time and resources to develop trusting relationships and deliver culturally and linguistically appropriate patient-centered care; 3) choose metrics based on evidence-based medicine for the populations and not just on measures that will save money; 4) utilize risk adjustment mechanisms to take into account the challenges that clinics and providers face whose patients have high SDOH burden; and 5) reward relative improvement in quality scores rather than attainment of an absolute threshold number.

Finally, true improvements in healthcare quality measures cannot be achieved solely with medical actions inside clinics. Quality health requires a universal health care plan, or innovative or inclusive processes and payment mechanisms so that basic medical care is available to everyone. Quality health requires societal and community actions outside of the clinics, as that is where the societal inequities are influencing health. The government needs to recognize this and work to address health at the societal level, and not attempt to hold clinics solely responsible for societal needs.

## Discussion

This qualitative study of individual interviews and focus groups with primary care providers (PCPs) who had worked both in safety net clinics (SNCs) and non-safety net clinics (NSNCs) in metropolitan Minneapolis-St Paul Minnesota reveals PCPs’ critique of the current quality metric system and processes to align payment with scores on quality metrics. Participating PCPs see that quality measurements do not fairly identify which clinics provide superior care. They assert that current quality metrics, developed from inequitable evidence and selection biases, reflect and intensify social disparities. This view illustrates one way in which the American medicine system is influenced by structural racism.

Participants perceive the measures as more influenced by patient and clinic factors than by clinician factors. (Theme #1). SNCs have worse quality scores given that they serve patients with high SDOH burdens and have low resources to respond to multiple patients’ needs. In contrast, NSNCs have higher scores because they serve patients with low SDOH burdens and have more resources to create processes to prioritize what is measured. In their opinions, the current system of quality measurement does not truly measure quality health care as patients and clinicians define quality health care (Theme #2). They are not alone in these assessments. Significant disparities in health care have been correlated to patients’ social-structural determinants of health, which are out of the control of clinicians [32]. One qualitative study of PCPs’ early reactions to accountable care organizations (ACOs) processes found a similar concern about the challenges of being held responsible for quality measure results that are affected by societal factors that are beyond their control (i.e., patients’ SDOH) [24]. Likewise, SNCs serving patients with high burdens of SDOH are more likely to be financially punished by quality-based payment systems [33], which would leave them with even fewer resources to focus on both quality metrics and their patients’ social complexities. Furthermore, the inequitable selection biases that have designed and chosen these measures have resulted in metrics that may not be as important to vulnerable populations served by SNCs, [11, 12]; other health issues as defined by communities themselves may be more valuable, such as mental health and substance use [1].

Overall, these PCPs criticize the current quality metric systems as being a product of, and embedded in, social and structural inequities of the American health care system, and warn that tying financial payments to these inequitable processes would exacerbate current health disparities (Theme #3). Similar PCP perspectives about aligning quality metrics and payment have been described (24]. Evidence for these concerns is seen in large studies indicating that physician participation in ACOs (one of the most popular delivery system reforms utilizing quality metrics) is less prevalent in disadvantaged communities [34] and that those organizations serving minority populations perform worse on quality metrics [6]. Similar concerns that aligning payment with quality measures will not redress societal inequities and health care disparities have been documented in interviews with a broad range of health care professionals, including clinicians [25, 26], medical directors [35–37], health care administrators [25, 35], and hospital executives [36–39] as well as in a systematic review about financial reimbursement for hospitals [40]. If ACOs and value based purchasing, aimed at improving quality and reducing spending, are less effective in diverse, vulnerable, or socio-economically disadvantaged populations, they will not be successful in achieving the desired results and there is potential to actually exacerbate existing disparities by connecting payment with current quality metrics [6, 34]. These published studies corroborate the PCP participants’ perspectives that the prior quality metric design and implementation approaches have historically not supported, and will not lead to, improved quality health care in all populations, and ultimately should not be reinforced with an inequitable P4P system.

These clinicians want a just and equitable health care system, echoing calls for health equity from around the world [41], the United States [42, 43]*,* and Minnesota [44]. Hardeman et al. [45], from Minnesota, argue that achieving health equity requires that healthcare professionals use their power to explore, understand and respond to the underlying structural racism that is undergirding the inequitable system. They build on Jones [46] to state: “Structural racism — a confluence of institutions, culture, history, ideology, and codified practices that generate and perpetuate inequity among racial and ethnic groups — is the common denominator of the violence that is cutting lives short in the United States.” (45, page 2113) While several participants use the term “SDOH” to describe the challenges that patients from disparate neighborhoods and communities face in obtaining quality health care, they acknowledge the structural racism that underlies these SDOH [45–48]. This recognition leads to expanding the term from SDOH to Structural/Social Determinants of Health Inequities (S-SDOH) in order to directly acknowledge the structural racism that underlies all of these inequitable social determinants of health [49].

### Limitations

As with all qualitative research, the limited number of people interviewed limits the generalizability of the results to other populations or locations. In addition, the participants’ experience was focused on the metropolitan area of Minneapolis-Saint Paul, but did not represent all of the major NSNCs or SNCs in the metropolitan area in Minnesota, or all systems throughout Minnesota. Also, participants’ temporal experiences of SNCs and NSNCs mean that they were not comparing current quality practices within these clinical systems. Because more participants were currently working in SNCs may indicate underlying biases towards SNCs over NSNCs. A quantitative survey with open-ended questions of clinicians currently working in NSNCs and SNCs may address some of these limitations. Nonetheless, participants’ assessments of the difference between SNCs and NSNCs are similar, and hence are summarizable, and provide a cohesive view based on their experiences.

## Conclusion

These PCPs in Minnesota, USA who have worked in both safety net (SNC) and non-safety net clinics (NSNCs) perceive that 1) current clinic quality scores are influenced more by patients and clinic systems than by clinicians; 2) current quality measures are not measuring quality healthcare; and 3) current quality measures are a product of and embedded in social and structural inequities in the American health care system, which should not be reinforced with financial payments such as current P4P or VBP, as aligning payment with the current quality metrics could perpetuate and exacerbate the existing social inequities and health disparities. They recommend that a new comprehensive approach to measuring and reimbursing quality needs to be designed that truly measures quality healthcare, is equitable and fair, and does not exacerbate the current inequities in the American healthcare system.

The National Quality Forum’s Roadmap for Promoting Health Equity and Eliminating Disparities, which was published after these interviews, is an approach that is consistent with the PCPs perspectives and recommendations, as it illustrates how quality metrics could be used to improve health equity [50]. One main NQF proposed strategy to achieve equity (“Incentivize the Reduction of Health Disparities and Achievement of Health Equity”) is directly relevant to PCP’s concerns for P4P and VBP. Several final recommendations: “Redesign payment models to support health equity”; “Support closing disparities by providing additional payments to providers who care for patients with social risk factors”; “Ensure organizations disproportionately serving individuals with social risk can compete in value-based purchasing programs”; and “Fund care delivery and payment reform demonstration projects to reduce disparities” [50, page 3] are consistent with the study PCPs recommendations. Policymakers should listen to PCP’s perspectives and work with PCPs to create a fairer quality metric system with an equitable quality-payment approach that does not perpetuate the inequitable system.

## Additional file

Additional file 1: Key Informant and Focus Group Questions. (DOCX 110 kb)

## Abbreviations

ACO: Accountable care organizations
AMP: Andrew M Pattock
DHS: Department of Human Services
DJS: David J Satin
FG: Focus group
KACP: Kathleen A Culhane-Pera
KI: Key informants
LMO: Luis Marty Ortega
LSO: Lynne S Ogawa
MDH: Minnesota Department of Health
MS: Michael Scandrett
MST: Mai See Thao
N: Number
NSNCs: Non-safety net clinics
P4P: Pay for performance
PCP: Primary care provider
PharmD: Doctor of Pharmacology
QMEP: Quality Measurement Enhancement Project
RN: Registered nurse
SDOH: Social-structural determinants of health
SES: Socio-economic status
SLP: Shannon L Pergament
SNC: Safety net clinics
VBP: Value-based payment

## Footnotes

1 QMEP’s projects have included improving methods of collecting data on SDOH factors, identifying the relationships between these factors and quality scores, adjusting or explaining quality scores in light of these relationships, and developing new or modified quality measures related to provider performance in identifying and addressing patients’ SDOH risk factors to improve health and treatment outcomes.
