# Supplementary material for: Primary care clinicians’ perspectives about quality measurements in safety-net clinics and non-safety-net clinics
Source: Int J Equity Health. 2018 Nov 7;17:161. doi: 10.1186/s12939-018-0872-3 (PMC6222992; doi:10.1186/s12939-018-0872-3)
Supplement: Supplementary file 1 — Key Informant and Focus Group Questions. (DOCX 110 kb) [file 12939_2018_872_MOESM1_ESM.docx]

**Additional file 1: Key Informant and Focus Group Questions**

**I. Compare and contrast data at two clinics from locations worked- one safety-net clinic and one non-safety-net clinic.**

A. Their non-safety net primary care clinic quality scores

From your experience, what do you think is contributing to this clinic’s scores?
Follow-up probes: Patient factors. Clinic factors. Clinician factors.

B. Their safety net primary care clinic quality scores

From your experience, what do you think is contributing to this clinic’s scores?
Follow-up probes: Patient factors. Clinic factors. Clinician factors.

C. Comparing the two clinics:

1. What differences do you think are making one clinic’s overall scores lower, and one clinic’s overall scores higher?

Follow-up probes: Patient factors. Clinic factors. Clinician factors.
 2. What do you think are the most important factors?

**II. Impact of quality measures on health care**

A. Personal experience
 1. What do these quality measures mean to you?
 2. How have these quality measures affected your work, or your health care system?
 3. How have these quality measures affected your interactions with patients, and your medical decisions?
 4. How have these quality measures affected your satisfaction with your work/ work place?

B. Future payment
1. In the foreseeable future, clinic/ provider payments could be based on/ influenced by quality measure results. What do you think about this?
 2. What do you think about creating a combined quality and payment system that accounts for patients’ characteristics, such as social determinants of health? Do you have ideas about how to achieve this?

**III. Patient perspectives about quality**

A. Patient ‘s view of quality.
 1. What do you think patients think about quality? What does quality mean to them?

2. What quality outcome measures are most important to patients?

3. Do patients distinguish between measures of health care process versus disease outcome
B. Clinic resources/ services desired

1. What resources or services do they say they need, in order to improve their health?
 2. Do you think these services would simultaneously improve clinic’s healthcare quality scores?

IV. **An improved future system of quality**

A. If you were to design your own system, what quality measures would be important to you? Why?
 B. What changes would you make to the current healthcare quality measures/ measurement approach?
